# Advanced Sensing and Safety Control for Connected and Automated Vehicles

**DOI:** 10.3390/s23021037

**Published:** 2023-01-16

**Authors:** Chao Huang, Yafei Wang, Peng Hang, Zhiqiang Zuo, Bo Leng

**Affiliations:** 1 Department of Industrial and Systems Engineering, The Hong Kong Polytechnic University, Hong Kong 999077, China; 2 School of Mechanical Engineering, Shanghai Jiao Tong University, Shanghai 200240, China; 3 Department of Traffic Engineering, Tongji University, Shanghai 201804, China; 4 School of Electrical and Information Engineering, Tianjin University, Tianjin 300072, China; 5 School of Automotive Studies, Tongji University, Shanghai 201804, China

The connected and automated vehicle (CAV) is a promising technology, anticipated to enhance the safety and effectiveness of mobility. Advanced sensing technologies and control algorithms, working to acquire environmental data, analyze data, and regulate vehicle movements, are key functional components of CAVs. In recent years, the creation of innovative sensing technologies for CAVs has gained substantial attention. CAVs can now interpret sensory data to more accurately detect impediments, locate their locations, navigate autonomously in a dynamic environment, and communicate with other nearby vehicles. This has been made possible by advances in sensing technology. Additionally, by utilizing computer vision and other sensing techniques, in-cabin persons’ bodily movements, facial expressions, and even mental states can be identified.

This Special Issue on *Sensors* aims to report on some of the recent research efforts on this increasingly important topic. The 11 accepted papers in this Issue cover vehicle position estimation [1], 3D Object Detection [2], pedestrian state sense [3], trajectory prediction [4], criticality assessment [5], active fault-tolerant control for actuator failure [6], decision-making in the scenario of highway driving out of the ramp [7] and uncertain interactive traffic scenarios [8], RRT-based path planning algorithm [9], C/GMRES motion planning algorithm [10], and lane-keeping controller design [11]. In this introduction, a brief description of the content of each contribution forming the Special Issue is provided. 

Localization, or a vehicle’s capacity to ascertain its position and orientation, is one of the crucial capacities needed to accomplish fully autonomous driving. [1] The first article describes a general, modular image processing pipeline that improves the robustness and accuracy of feature-based VO techniques, allowing for better pose estimation. Contrast-limited adaptive histogram equalization (CLAHE), square covering (SSC), and angle-based outliers rejection (AOR) are the three stages of the proposed pipeline. Each level deals with a different problem related to posing estimate errors. In order to improve the performance of any feature-based algorithm, the proposed pipeline is designed to be generic and modular so that it can be incorporated into any existing method. The proposed pipeline offers a large improvement in VO accuracy and resilience, with just a slight increase in processing time, according to the quantitative and qualitative results.

Ref. [2] proposes an easy-to-use technique for up-sampling low-resolution point clouds, a process which improves the accuracy of 3D object detection by reconstructing objects rendered in sparse point cloud data into more dense data. First, 4-Chs is used to transform the 3D point cloud dataset into a 2D range image. In order to preserve the forms of the original object throughout the reconstruction, the interpolation on the empty space is calculated based on both the pixel distance and the range values of six neighbor points.

In [3], a novel pedestrian status-detecting approach, based on multi-sensor fusion, is developed for automated patrol vehicles. First, the 2D and 3D pedestrian data are acquired through YOLO V4 and Euclidean clustering algorithms, respectively. A novel multi-sensor fusion method, based on the R-Tree algorithm, is then designed to detect the pedestrian and the body temperature is detected based on thermal imaging. The experimental results on an automated patrol vehicle indicated that the single-sensor algorithm’s pedestrian detection results could be improved by more than 17% and that the multi-layer fusion method provided more understandable density estimation results. 

A multi-head attention-based LSTM sequence-to-sequence model is designed in [4] to predict the future trajectories of the ego vehicle’s surrounding vehicles. The social and temporal interaction are successfully extracted by the authors using a multi-head transformer method, and each one is then encoded into the input as hidden information using an LSTM encoder in the encoder module. The hidden information is decoded using the decoder module, which uses an additional multi-head attention layer to increase the accuracy of the subsequent decoding. The proposed model, according to ablative studies, resolves the cumulative error brought on by the transformer’s autoregressive decoding behavior. The visualization outcomes demonstrate the model’s robustness in challenging congested conditions.

In [5], a novel risk assessment method, based on the time-to-react measurement, is proposed to determine overall criticality. The novelty of this method lies in the introduction of variable threshold values which are converted into the time domain from minimal safety distance metrics and acceleration values for criticality level determination. The designed variable criticality threshold closes a research gap by accounting for the kinematic relationships between vehicles and, as a result, the variable nature of criticality. The proposed method is proven to have the advantages of simplifying the consideration of the surrounding ego vehicles and evaluating possible evasive actions.

In [6], an active fault-tolerant control (AFTC) system is designed for the longitudinal motion control of autonomous mobile robots (AMRs) in the condition of braking failure of actuators. Specifically, a velocity-tracking controller is designed, with integrated feedforward and feedback control for normal longitudinal driving control. Once a key actuator failure is detected, the driving/braking torques of the remaining normal actuators are then reallocated based on the weighted least-squares (WLS) method for failure compensation. The simulation results show that the proposed AFTC algorithm can deal with the braking failure of IWMs and realize braking energy recovery at the same time.

Ref. [7] proposes a generalized single-vehicle-based graph neural network reinforcement learning (SGRL) algorithm that incorporates interactive information between agents in the environment into the decision-making process of autonomous driving. The proposed SGRL algorithm uses the training approach for a single agent, constructs a clearer incentive reward function, and greatly increases the dimension of the action space over the conventional deep neural network (DQN) algorithm. The simulation results show that the proposed SGRL algorithm excels in terms of network convergence, decision-making impact, and training effectiveness.

Similar to [7], a reward function matrix for training different decision-making modes is provided in [8], with emphasis placed both on decision-making styles and, additionally, on rewards and penalties. A decision-weighted coefficient matrix, an incentive–punishment-weightedcoefficient matrix, and a reward–penalty function matrix are all included in the proposed reward function matrix. The simulation results demonstrate that the proposed reward function can significantly increase the algorithm’s stability and speed of convergence.

Ref. [9] proposes using an improved heuristic Bi-RRT algorithm to obtain a smooth and asymptotically optimal path, with continuous curvature possessing high efficiency and accuracy in an uncertain dynamic environment. The consideration of the driver’s driving habit and the obstacle-free direct connection mode of two trees, as well as the introduction of the greedy step size and the design of the path reorganization, can expand the node more effectively, make the path smooth, and ensure the ride comfort of the vehicle. The simulation results show that the proposed algorithm can generate the smoothest path and take the shortest time compared with the current studies.

In [10], a computationally efficient motion planning method that both considers traffic interactions and accelerates calculation is proposed. A nonlinear predictive controller is designed to dynamically generate a path by considering the predicted trajectories of other traffic participants. In addition, the C/GMRES algorithm is used to accelerate the calculation of trajectory generation. The simulation experiments show that the proposed path planning algorithm can enable greater rationality of movement planning.

Ref. [11] proposes a cooperative control strategy for lane-keeping by integrating driving monitoring, a variable level of assistance allocation, and human-in-the-loop control. The relationship between lateral acceleration, road curvature, and the observed maximum driver torque is used in the first stage of this research to identify a time-varying physical driver loading pattern. An adaptive driver activity function is then developed to simulate the amount of support needed for the driver in the following stage. Additionally, this is combined with a monitored driver state that signals the driver’s mental loading. A unique higher-order sliding mode controller is developed to maintain closed-loop stability in order to seamlessly switch control between different modes, based on the generated amounts of assistance. Additionally, the conflict is reduced by using a new sharing parameter that is proportionate to the torques originating from both the driver and the autonomous controller.

In summary, there is a huge potential for the application of CAV in areas of traffic perception, decision-making, and driving safety improvement. This Special Issue contributes to this by proposing solutions to the general problems of state estimation [1], objective detection [2,3], trajectory prediction [4], driving safety improvement [5,6], decision-making [7,8], path planning [9,10] and vehicle motion control [11], which largely represent the theoretical challenges and practical interest of this research topic. Finally, we wish to thank the authors, reviewers, and journal staff for their commitment and effort, without which we could not have completed this Special Issue on time. We hope that readers enjoy reading the articles and that the published works contribute to the progression of connected and automated vehicles.
